# Beliefs and practices of patients with advanced cancer: implications for communication

**DOI:** 10.1038/sj.bjc.6601950

**Published:** 2004-06-22

**Authors:** G F Beadle, P M Yates, J M Najman, A Clavarino, D Thomson, G Williams, L Kenny, S Roberts, B Mason, D Schlect

**Affiliations:** 1 Division of Translational and Clinical Research, Queensland Institute of Medical Research, PO Royal Brisbane Hospital, Herston, Queensland 4029, Australia; 2 Wesley Cancer Care Centre, Wesley Hospital. Chasely Street, Auchenflower, Queensland 4066, Australia; 3 School of Nursing, Queensland University of Technology, School of Nursing, Victoria Park Road, Kelvin Grove, Queensland 4059, Australia; 4 Department of Anthropology and Sociology, University of Queensland, St Lucia, Queensland 4067, Australia; 5 Centre for Health Promotion and Cancer Prevention Research, University of Queensland, St Lucia, Queensland 4067, Australia; 6 South Brisbane Oncology Research Unit, Princess Alexandra Hospital, Ipswich Road, Woolloongabba, Queensland 4102, Australia; 7 Australian Centre for International and Tropical Health and Nutrition, University of Queensland, St Lucia, Queensland 4067, Australia; 8 Queensland Radium Institute, Royal Brisbane Hospital, Herston Road, Herston, Queensland 4029, Australia

**Keywords:** belief in curability, need for control, communication, advanced cancer

## Abstract

The aim of this study was to investigate the beliefs that patients with advanced cancer held about the curability of their cancer, their use of alternatives to conventional medical treatment, and their need to have control over decisions about treatment. Of 149 patients who fulfilled the criteria for participation and completed a self-administered questionnaire, 45 patients (31%) believed their cancer was incurable, 61 (42%) were uncertain and 39 (27%) believed their cancer was curable. The index of need for control over treatment decisions was low in 53 patients (35.6%) and high in only 17 patients (11.4%). Committed users of alternatives to conventional medical treatments were more likely to believe that their cancer was curable (*P*<0.001) and to have a higher need for control over decisions about treatment (*P*<0.004). The mean need for control scores were highest in patients who believed that their cancer was curable, or who were uncertain about the curability of their cancer, but who acknowledged that their oncologist had reported that the cancer was incurable. The diverse beliefs, attitudes and actions of these patients were consistent with a range of psychological adaptions to a life-threatening illness, some realistic and others illusory. Illusory responses influence what communication can achieve in daily oncology practice.

Accurate communication of information underpins the contemporary Western medical values of truth telling and patient participation in decision making, as well as the core ethical, cultural and legal values of informed consent. Recent research has placed a considerable emphasis on effective communication of bad news (Fallowfield, 1993; Sell *et al*, 1993; Ptacek and Eberhardt, 1996) and numerous approaches have been recommended to evaluate and improve communication (Fallowfield, 1993; McConnell *et al*, 1999; Schofield *et al*, 2001). Yet relatively little attention has been paid as to what patients believe about what they are told and how they respond to bad news.

In advanced cancer, there is growing evidence that some patients have unrealistic expectations of treatments of minimal (Slevin *et al*, 1990) or unproven efficacy (Bagenal *et al*, 1990; Daugherty *et al*, 1995; Miller *et al*, 1998), including a belief that their cancer is curable (Slevin *et al*, 1990; Yates *et al*, 1993; Butow *et al*, 1999; Richardson *et al*, 2000). In a secondary analysis of data that evaluated the beliefs and attitudes of patients about their diagnosis, prognosis, satisfaction with and use of treatments in advanced cancer, we showed that positive illusory responses, characterised by a belief in curability, a strong will to live and a committed use of alternatives to conventional medical treatments were associated with better quality of life (Beadle *et al*, 2004). The purpose of the current analysis is to evaluate the relationship between the beliefs held by these patients about the curability of their cancer and their need to have control over decisions about treatment.

## MATERIALS AND METHODS

### Participants

The details of this prospective cross-sectional study have been published elsewhere (Yates *et al*, 1993; Beadle *et al*, 2004) and its intent was to capture the immediate views of patients about their attitudes and beliefs following a diagnosis of advanced cancer. In summary, eligible patients had locally advanced or metastatic cancer, measurable or evaluable disease, ambulatory status (performance status ECOG 0–3), an estimated survival between 3 months and 2 years, and a time interval since last outpatient review of less than 3 months. All were potential candidates for active treatment of their cancer as an outpatient, and 90% were having systemic and/or radiation treatments at the time of this study. The clinical standard of practice was to inform all patients about the extent of their cancer, its incurability, the expectations of a limited lifespan, the palliative goal of systemic and radiation treatment, the response to treatment and their overall progress. The study was approved by the Ethics Committees of the relevant institutions and all participants provided written informed consent after explanation of the purpose of the study.

### Questionnaire

Of five items that examined attitudes and beliefs about treatment decisions, two statements specifically asked patients about their need to have control over decisions about treatment. On a scale of 1 (very true) to 4 (not true at all), patients were asked to report their need for control over decisions about treatment. One item was reverse coded and the scores of both questions were added to create an index, with a potential range of 2 (low need for control) to 8 (high need for control).

Patients were also asked to indicate the degree to which they utilized and believed in the efficacy of alternatives to conventional medical treatments. Details of therapies and the practitioners who prescribed alternatives to conventional medical treatments are described elsewhere (Yates *et al*, 1993) in a larger sample of patients. Therapies included vitamins and tonics, methods of meditation and relaxation, special diets, faith healing, herbal remedies, and methods of detoxification and immune stimulation. A quantitative analysis of questions looking at the use of, and commitment to, these alternatives was followed by a detailed qualitative assessment of patients' beliefs and attitudes. Committed users were characterised by a strong adherence to the use of alternatives and a strong belief that alternatives were a core part of their treatment. A total of 33 patients fulfilled the criteria while the remaining 116 patients did not utilise alternatives or made only minimal alternative changes to their lifestyle.

The concept of belief in curability of cancer was assessed by three items. Two items asked patients about their belief in the curability of their cancer and how long they expected to survive, and one question asked their recollection of the report by their oncologist about the curability of their cancer (Table 1

**Table 1:** Index of belief in curability

I believe that my cancer:
1. Is curable
2. Is not curable
3. Don't know

Regardless of what your doctors have told you, how long do you expect to survive?
1. My condition is curable
2. One year or more
3. Some months
4. Some weeks
5. No idea/don't know

My doctor says my cancer
1. Is curable
2. Is not curable
3. I am not certain what my doctor has told me about cure
4. My doctor has said nothing to me about cure

). The respondents' beliefs about curability and their recollection of what their oncologist had reported were each categorised into three groups: curable, uncertain and incurable.

### Statistical analysis

Data were analysed using the statistical package SPSS. Kendall Tau_b_ correlation coefficients were calculated among items that addressed a particular concept. Associations between categorised variables were examined with *χ*^2^-tests. Variation among subgroups in mean need for control scores was examined using analysis of variance (three subgroups) or *t*-tests (two subgroups).

## RESULTS

### Participants

During the 10-week period of this prospective study, 178 consecutive patients potentially fulfilled the criteria for participation. After exclusions, 149 patients fulfilled the criteria for participation and completed the questionnaire.

### Belief in curability and committed use of alternatives

In this study, 39 patients (27%) believed that their cancer was curable, 61 (42%) were uncertain about the curability of their cancer, and 45 (31%) believed that their cancer was incurable. Table 2

**Table 2:** Committed use of alternatives to conventional medical treatment and the distribution of belief in curability

		**Committed use**	**No/minimal use**
		**(*n*=33)**	**(*n*=116)**
**Belief in curability^a^**	**Number (%)**	**Number (%)**	**Number (%)**
Curable	39 (27)	17 (52)	22 (19)
Uncertain	61 (42)	10 (30)	54 (47)
Incurable	45 (31)	4 (18)	40 (34)
			*X*=13.37 (df=2) *P*<0.001

a Four items incomplete.

shows the distribution of belief in curability according to the committed use of alternatives to conventional medical treatments. Committed users of these alternatives were more likely to believe that their cancer was curable than the remaining patients (*P*<0.001).

### Need for control over treatment decisions

The statements of need for control over decisions about treatment and the distribution of scores are shown in Table 3

**Table 3:** Need for control over treatment decisions

**Statements**		
**I leave it up to my doctor to decide what is best for my cancer.**		
**I need to have control over decisions made about my cancer treatment**		
**Scores**	**Number**	**Percentage**
2	32	21.5
3	21	14.1
4	24	16.1
5	42	28.2
6	11	7.4
7	6	4.0
8	11	7.4
Missing	2	1.3
Total	149	100

. The two items comprising this index had a Kendal Tau_b_ of 0.32 (*P*<0.001). A total of 53 patients (35.6%) had a very low need for control (scores 2,3), with 119 (79.9%) scoring at or below the mid-point of 5. Only 17 patients (11.4%) had a high need for control over decisions about treatment (scores 7,8).

### Need for control and belief in curability

Table 4

**Table 4:** Mean need for control scores, patients' beliefs in curability, and use of alternatives to conventional medical treatment

	**Number**	**Need for control mean score**
*Committed use of alternative treatment*^a^		
Committed	33	6.09
No/minimal	114	3.76
	*t*(1,145)=64.55, *P*<0.004	
		
*Belief in curability*^b^		
Curable	38	4.79
Uncertain	63	4.16
Incurable	45	4.02
	*F*=(2,143)=2.24, *P*=0.11	
Curable	38	4.79
Uncertain & Incurable	108	4.10
	*t*(1,144)=4.35, *P*=0.04	

a Two items missing.

b Three items missing.

shows the mean need for control scores distributed according to the use of alternatives to conventional medical treatments, and belief in curability. Parametric statistical tests were used for these analyses since the normality assumptions for the need for control scale were satisfied. The mean need for control scores were significantly higher in patients who were committed users of alternatives than the mean scores of those patients who did not use alternatives or who made only minimal changes to their lifestyle (*P*<0.004). There was a trend for patients who believed that their cancer was curable to have a higher mean need for control score than patients who were uncertain about the curability of their cancer or who did not believe that their cancer was curable (*P*=0.11 for the three groups; *P*=0.04 when the group who believed in curability was compared with the remaining groups).

Table 5

**Table 5:** Belief in curability^a^ and need for control scores^b^

	**Recollections of oncologists' reports of curability**			
	**Curable**	**Uncertain**	**Incurable**	
**Patients beliefs in curability**	***X̄* (*N*)**	***X̄* (*N*)**	***X̄* (*N*)**	***P***
Curable	3.87 (16)	5.25 (8)	5.47 (15)	0.06 f(2,35)=3.14
Uncertain	4.00 (12)	4.32 (25)	4.00 (24)	NS
Incurable	— (0)	4.87 (8)	3.84 (37)	NS
*P*	NS	NS	0.015	

a Four items incomplete group.

b *X̄* is the mean score for each group; (*N*) is the number of patients in each.

shows the mean need for control scores distributed according to patients' beliefs in curability and their recollection of the reports by their oncologists. Of the 45 patients (31%) who believed that their cancer was incurable, 37 recollected a report of incurability by their oncologist and eight were uncertain of their oncologist's report. For this group, a recollection of incurability generally corresponded with the intended communication by the treating oncologist. Of the remaining 100 patients, 39 (27%) believed their cancer was curable and 61 (42%) were uncertain about curability. Of these patients, 28 recollected a report of curability of their cancer by their oncologist and 33 were uncertain about the report by their oncologist. However, 39 patients who believed their cancer was curable or who were uncertain about its curability acknowledged a report of incurability by their oncologist. The mean need for control scores were highest in those patients who recollected a report of incurability by their oncologist but believed their cancer was curable (5.47) or were uncertain about the curability of their cancer (5.25). The mean scores of patients who believed their cancer was curable differed significantly from the mean scores of patients who believed their cancer was incurable and who recollected a reported incurability by their treating oncologist (*P*=0.015).

## DISCUSSION

Studies of disclosure of bad news recognise the need to communicate complex information effectively in an emotionally charged consultation. At odds are the practitioner's duty to truth telling and the patient's cognitive and emotional appraisal of bad news. Techniques for delivery of bad news are well documented in oncology (Sell *et al*, 1993; Girgis and Sanson-Fisher, 1995), but studies also recognise that information can be misunderstood or forgotten (Ptacek and Eberhardt, 1996; McConnell *et al*, 1999; Schofield *et al*, 2001). The use of audiotapes in medical consultations is a logical antidote to misunderstanding (Hogbin and Fallowfield, 1989; Damian and Tattersall, 1991; Deutsch, 1992; Dunn *et al*, 1993; McHugh *et al*, 1995) but, for patients with a poor prognosis, bad news can increase psychological distress (McHugh *et al*, 1995) and the potential for maladaptive responses. To date, most studies have focused on initial contact with bad news but not the subsequent coping patterns by patients. In one qualitative study, however, patients with small-cell lung cancer adapted to bad news by exhibiting false optimism during the course of their illness (The *et al*, 2000). In the current study, only 45 patients (31%) believed their cancer was incurable and this belief correlated with a recollection of a report of incurability by their oncologists in 37 cases. By contrast, 39 patients (27%) believed their cancer was curable, a result similar to that reported in other patients with metastatic cancer (Mackillop *et al*, 1988), while 61 patients (42%) were uncertain about the curability of their cancer. While inadequate communication and false optimism are plausible explanations of the discrepancies between patients' beliefs and their recollections of the reports by their oncologists, 39 (36%) of the 108 patients who believed their cancer was curable or were uncertain about its curability acknowledged a report of incurability by their oncologist. For these 39 patients, communication was adequate but at odds with their own beliefs.

In addition to assessing patients' beliefs about curability, this study evaluated the actions patients took to influence their outcome. An index of need for control over decisions about treatment was skewed with 36% scoring at the lower end of the range and only 11.4% at the upper end. Patients who were committed users of alternatives to conventional medical treatments, however, had a higher need for control scores (Table 4) and were more likely to believe that their cancer was curable (Table 2) than the remaining patients. Furthermore, the scores were highest in those patients who acknowledged a report of incurability by their oncologist but who believed their cancer was curable or were uncertain about curability (Table 5).

In recent years, increasing importance has been placed on the development of methods to improve comprehension about the likely value of treatments (Pater *et al*, 1994; Llewellyn-Thomas, 1995; O'Connor, 1995; Richardson and Detsky, 1995). However, studies also show that patients are prepared to accept a lower level of benefit from treatment than healthy surrogates (Sackett and Torrance, 1978; Boyd *et al*, 1990; Llewellyn-Thomas *et al*, 1991; Coates and Simes, 1992; Llewellyn-Thomas *et al*, 1992). These results suggest that illness-related behaviour is more than the sum of communicated information. In the current study, an assessment of belief in curability showed discrepancies between the intent of the oncologists to present accurate information about prognosis, and the beliefs of some patients. While inaccurate or falsely optimistic communication are possible explanations of some discrepancies, the range of beliefs and attitudes suggests that at least some patients reorientated the experience of adverse information to a positive imaginative outcome. In particular, some patients who exhibited a high need for control were not passive recipients of information but actively shaped information and undertook strategies to support positive illusory beliefs.

Illusions contribute to the diversity of human responses to both information and circumstance, and positive illusions are well recognised in both health and illness, sometimes with beneficial outcomes (Taylor and Brown, 1988). The extent to which some patients reshape information to meet their own conative, cognitive and affective dispositions influences the intended goals of both communication and informed consent in oncology practice, and also plausibly explains their vulnerability to biased information.
